# Clinicopathological investigation of secretory carcinoma cases including a successful treatment outcome using entrectinib for high-grade transformation: a case report

**DOI:** 10.1186/s12920-022-01155-6

**Published:** 2022-01-06

**Authors:** Kensuke Suzuki, Hiroshi Harada, Masayuki Takeda, Chisato Ohe, Yoshiko Uemura, Akihiko Kawahara, Shunsuke Sawada, Akira Kanda, Bhaswati Sengupta, Hiroshi Iwai

**Affiliations:** 1 Department of Otolaryngology, Head and Neck Surgery, Kansai Medical University, 2-5-1, Shin-machi, Osaka 573-1010 Hirakata, Japan; 2 Department of Diagnostic Pathology and Cytology, Osaka International Cancer Institute, 3-1-69, Otemae, Chuo-ku, Osaka, 541-8567 Japan; 3 Department of Medical Oncology, Kinki University, 377-2, Ono-higashi, Osaka-Sayama, Osaka 589-8511 Japan; 4 Department of Pathology, Kansai Medical University, 2-5-1, Shin-machi, Hirakata, Osaka 573-1010 Japan; 5 Department of Diagnostic Pathology, Kurume University Hospital, 67 Asahi-machi, Kurume, Fukuoka 830-0011 Japan; 6 IVD Assay Development Department, ArcherDX, LLC, an Invitae Company, 2477 55th Street, Suite 202, Boulder, CO 80301 USA

**Keywords:** Secretory carcinoma, Salivary gland, High-grade transformation, Next-generation sequencing, *ETV6-NTRK3*, Entrectinib

## Abstract

**Background:**

Secretory carcinoma (SC) of the salivary gland is a recently described malignant tumor harboring characteristic *ETV6-NTRK3* gene fusion. SC generally has a favorable clinical course, and is currently regarded as a low-grade carcinoma. However, a small subset of SCs demonstrates aggressive clinical features with histologically high-grade transformed morphology, the molecular pathogenesis of which has not yet been elucidated. In this study, we performed a clinicopathological and molecular genetic study of patients with SC of the head and neck displaying various clinical characteristics to investigate the differences of pathological and molecular genetics between low-grade and high-grade components of SC.

**Case presentation:**

Three cases with SC of the head and neck, including a conventional low-grade SC and two high-grade transformed SCs are described. High-grade transformed SCs with histological features such as nuclear polymorphism, distinctive nucleoli and increased mitotic activity developed locoregional recurrence and distant metastasis. Immunohistochemical analysis revealed that low- and high-grade components showed different expression patterns for S-100 protein and mammaglobin, whereas all examined components were positive for p-STAT5. p53-positive cell population was markedly higher in one case with high-grade transformed SC. The proliferative activity of high-grade components was markedly increased, with the Ki-67 labeling index ranging up to 30–32%. A fluorescence in situ hybridization study with an ETV6 (12p13) break apart probe revealed split signals in the nuclei in all 3 cases. A targeted next-generation sequencing-based fusion assay demonstrated that all 6 clinical samples from the 3 patients showed the presence of the *ETV6-NTRK3* fusion transcripts. One patient with high-grade transformed SC showed a dramatic clinical response to the pan-TRK inhibitor, entrectinib, for the treatment of locoregional recurrence and pulmonary metastasis.

**Conclusions:**

High-grade transformed SC showed aggressive clinical and pathological features with increased Ki-67 labeling index. Molecular genetic study of gene rearrangement appears to be beneficial treatment as the presence of *ETV6-NTRK3* translocation may represent a therapeutic target in SC, particularly the high-grade transformed type.

**Supplementary Information:**

The online version contains supplementary material available at 10.1186/s12920-022-01155-6.

## Background

Secretory carcinoma (SC) of the salivary gland, also known as mammary analogue secretory carcinoma, is a recently described malignant tumor that harbors a characteristic chromosomal translocation t(12;15)(p13;q25) resulting in *ETV6-NTRK3* gene fusion [1]. Histopathologically, SC is a distinct entity, and histological in combination with appropriate immunohistochemical analysis is virtually sufficient for a diagnosis in most cases [2, 3]. However, several histopathological features of SC overlap with those of other salivary gland tumors, such as acinic cell carcinoma (AcCC), adenocarcinoma, not otherwise specified, and low-grade mucoepidermoid carcinoma [1, 4, 5]. The differentiation of SC from its mimickers is important due to the differences in their behavior and the possibility for molecular targeted therapy [6, 7]. Detection of ETV6 rearrangements by fluorescent in situ hybridization (FISH) or *ETV6-NTRK3* fusion by reverse transcriptase polymerase chain reaction (RT-PCR) in formalin-fixed paraffin-embedded (FFPE) specimens is relatively straightforward in technical terms and > 300 cases of SC have been reported since its initial description [8]. The clinical course of conventional SC is characterized by a moderate risk of local recurrence (15%) and lymph node metastases (20%) and a low risk of distant metastases (5%) [1, 9]. Clinical stage at the time of diagnosis is the most powerful predictor of prognosis. No other prognostic markers have been confirmed to be associated with clinical outcome [10].

High-grade transformation (HGT, originally referred to as “dedifferentiation”) is defined as the abrupt transformation of a low-grade (LG) or well-differentiated tumor into HG histology that lacks the original distinct pathological characteristics [11]. After the first report of a ‘‘dedifferentiated’’ acinic cell carcinoma (AcCC) of the parotid gland in 1988 [12], several authors described this phenomenon not only in AcCC, but also in other salivary gland carcinomas such as adenoid cystic carcinoma [13], epithelial-myoepithelial carcinoma [14], and polymorphous low-grade adenocarcinoma [15], all of which undergo ‘‘dedifferentiation’’ or HGT [11]. Therefore, this concept is now established for salivary gland neoplasms [11, 16]. Although SC is typically a LG malignancy with LG histopathologic features, some of SCs have been found to demonstrate HG histopathology with aggressive clinical features [10, 17]. Skálová et al. reported 3 cases of SC with HGT, and all patients died from the disease within 2 to 6 years after primary diagnosis [10]. HGT of salivary gland carcinomas is always associated with tumor progression. However, little is known about the molecular genetic events that regulate it [10]. In some previous reports, the involvement of several genes such as p53 and HER-2 has been reported to be involved in the HGT process of salivary gland tumors [11, 14, 18]. Nevertheless, the molecular mechanisms of HGT in SC remain unknown.

Recently, *ETV6-NTRK3* gene rearrangement has been identified as a therapeutic target [19, 20]. The safety and antitumor activity of entrectinib, a potent oral inhibitor of the tyrosine kinases TRKA/B/C, ROS1, and ALK, has been demonstrated for patients with advanced or metastatic solid tumors that harbor gene rearrangement, including NTRK1-3, ROS1, and ALK, regardless of histology in some clinical trials [6, 7]. In this study, we undertook a clinicopathological study of 3 patients with SC of the head and neck displaying various clinical characteristics, including one case of conventional LG, and two cases of HG-transformed SC to investigate the differences of histomorphological and immunohistochemical features between LG and HG components of SCs. For the purpose of analyzing the molecular pathogenesis of HGT in SC, we also performed RNA-based gene fusion analysis using next-generation sequencing (NGS). One patient with HG-transformed SC was enrolled in a phase II clinical trial (STARTRK-2) [21], and showed a dramatic clinical response to entrectinib without any serious adverse events.

## Case presentation

This study was approved by the Kansai Medical University Ethics Committee (approval # 2015103). Written informed consents were obtained from all of the participants in this study. For Case 3, written informed consent was obtained from his next of kin.

### Clinical findings

#### Case 1

A 37-year-old man presented with a 6-month history of a painless palpable mass in the left side of the parotid. On physical examination, the tumor was well circumscribed and movable, and was approximately 10 mm in diameter. Partial superficial parotidectomy was performed. The surgical margin was negative, and no adjuvant therapy was administered. No local recurrence or metastatic disease has been detected during a follow-up of 4 years.

#### Case 2

**Fig. 1:**
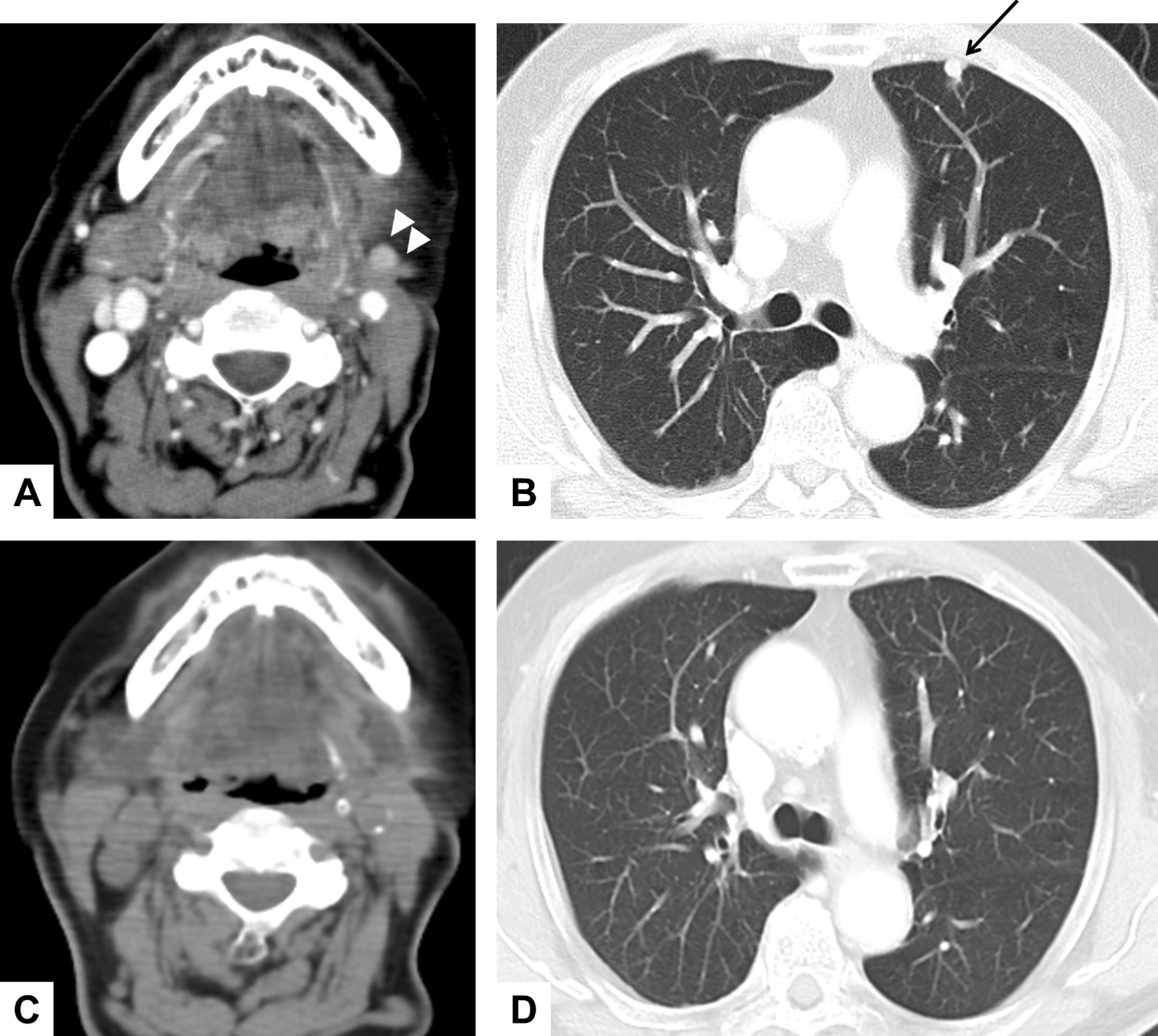
A durable complete response after entrectinib therapy in a SC patient with regional recurrence and pulmonary metastasis (Case 2). CT imaging of the patient before (**A** and **B**) and at 18 months after (**C** and **D**) entrectinib therapy are shown. Arrow heads indicate regional recurrence and the black arrow indicates pulmonary metastasis

This case was a 74-year-old man, the first cervical SC from an unknown primary site for whom we reported previously [22]. A CT scan at 18 months after surgery revealed a recurrent tumor in the left neck and pulmonary metastasis without any symptoms (Fig. 1A and B). Hybrid-capture-based next-generation sequencing of the initial tumor identified *ETV6-NTRK3* t(12;15)(p13.2;q25.3) rearrangement. He was then enrolled in a phase II clinical trial of the pan-TRK inhibitor, entrectinib (STARTRK-2). Entrectinib was administered orally at a dose of 600 mg once daily. CT imaging at 3 months revealed a dramatic, complete response in both the recurrent tumor in the left neck and the pulmonary metastasis. The treatment and response had been continued for 27 months at the time of writing (Fig. 1C and D). The patient noted general fatigue and nausea as treatment-related adverse events, but these were managed by entrectinib dose reduction.

#### Case 3

A 61-year-old male presented with a 4-month history of a painless lump in the right neck. He was referred to our department because of a diagnosis of salivary gland cancer and suspected residual tumor by excisional biopsy. Right partial parotidectomy and neck dissection were performed as additional surgery. The initial histopathological diagnosis was a mucinous adenocarcinoma as described in the 2nd edition of the World Health Organization Classification (1991), which was later recognized as a LG carcinoma. Two, 10, 14, 17, and 18 years after initial surgery, the patient experienced repeated recurrence, and further operations were performed (for the 1st to 5th recurrence, respectively). Two months after the final operation, he complained of dysphagia and hoarseness due to a right neck mass and laryngeal paralysis. CT scans showed locoregional recurrence in the ipsilateral neck and parapharyngeal space, and multiple metastases in the lung (6th recurrence). The patient died of neoplastic disease 18 years after primary surgery.

### Histologic and immunohistochemical findings

**Fig. 2:**
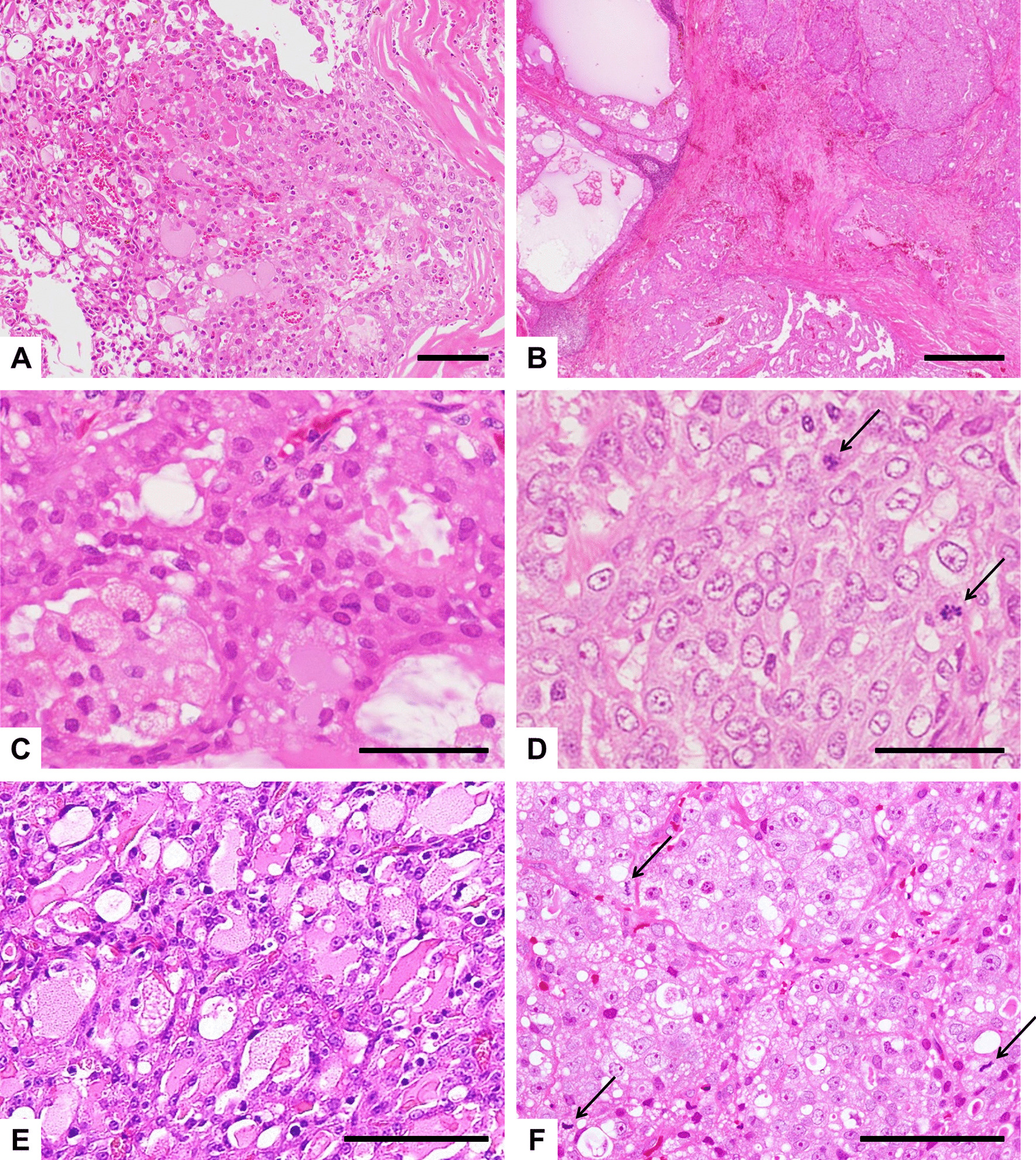
Histologic findings of SC cases. **A** Case 1; **B**–**D** Case 2; **E** and **F**, Case 3 (H&E*). **A** A tumor consisting mainly of a papillary-cystic growth pattern containing follicular structures. **B** Low-power view of the biphasic histology of the tumor comprising a HG carcinoma with a solid proliferative component (right portion), and a conventional LG SC with a papillary-cystic component (left portion). The LG component consisted of small, regular cells with an eosinophilic or vesicular cytoplasm (**C**), whereas the HG component consisted of enlarged, irregularly sized cells with prominent nucleoli, and several mitoses (arrows) can also be observed (**D**). **E** Histologic findings of the initial tumor exhibiting the characteristics of LG SC, consisting of tumor cells with unifying nuclear features accompanied with many microcystic structures containing eosinophilic secretions. **F** Histologic findings in the specimen from the 6th recurrence exhibiting the characteristics of a HG carcinoma, consisting of irregular, enlarged cells containing large pleomorphic nuclei and prominent nucleoli with high mitotic activity (arrows). Scale bars, 100 µm (**A**, **E**, **F**); 500 µm (**B**); 50 µm (**C**, **D**). *Hematoxylin and eosin

Microscopy images were acquired using BX53 and DP73 (Olympus, Tokyo, Japan). Histologically, the tumor in Case 1 was a conventional LG SC consisting mainly of a papillary-cystic growth pattern (Fig. 2A). The initial tumor in Case 2 was composed of 2 distinct sharply delineated carcinomatous components: One was a conventional LG SC of papillary-cystic type, and the other was a solid-type HG carcinoma (Fig. 2B). The LG component consisted of small and uniform neoplastic cells (Fig. 2C), whereas the HG component consisted of enlarged and irregularly sized cells with prominent nucleoli (Fig. 2D). For Case 3, we reviewed all samples from the initial tumor to the 6th recurrence. The initial tumor consisted mainly of a papillary-cystic growth pattern with microcystic structures of LG SC in appearance (Fig. 2E). The specimens from the 1st to the 3rd recurrences showed similar growth patterns as the initial tumor. In the tumor of the 4th and 5th recurrences, the histopathological features were apparently changed, as the tumor consisted mainly of a solid proliferative component (data not shown). For the biopsy specimen of the 6th recurrence, markedly enlarged and irregularly sized nuclei were observed and the nucleoli had become swollen and prominent (Fig. 2F). These findings with regard to cell morphology were completely different from the initial tumor, suggesting that HGT had occurred through the multiple recurrences. There was no evidence of nerve and vascular invasion in all LG components of 3 cases.

**Fig. 3:**
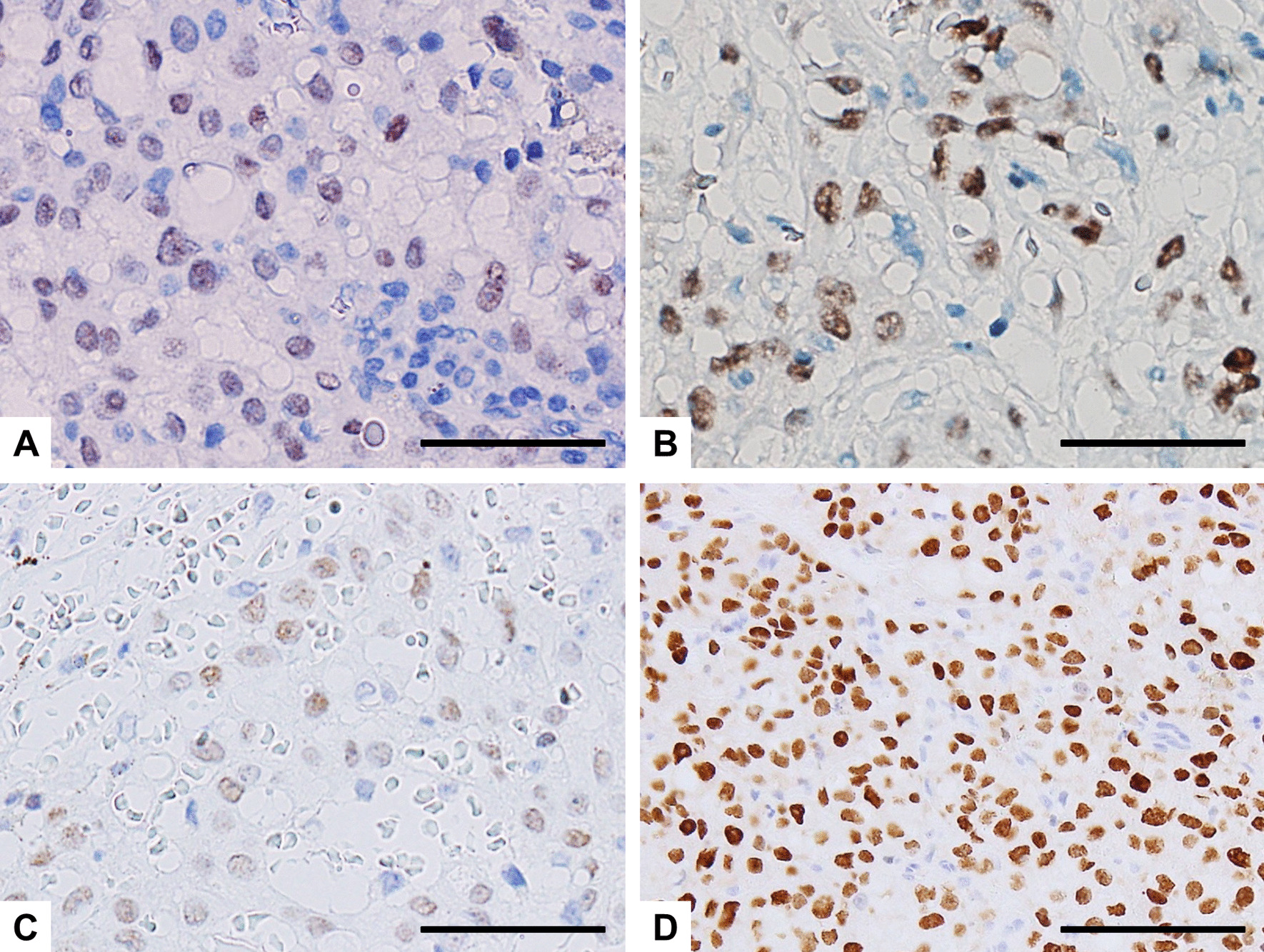
Immunohistochemical findings of p-STAT5 in SC cases. **A** Case 1; **B** Case 2; The initial tumor (**C**) and the specimen from the 6th recurrence (**D**) in Case 3. p-STAT5 is can be seen in the nuclei of all the samples examined. Scale bars, 50 µm (**A**–**C**); 100 µm (**D**)

**Fig. 4:**
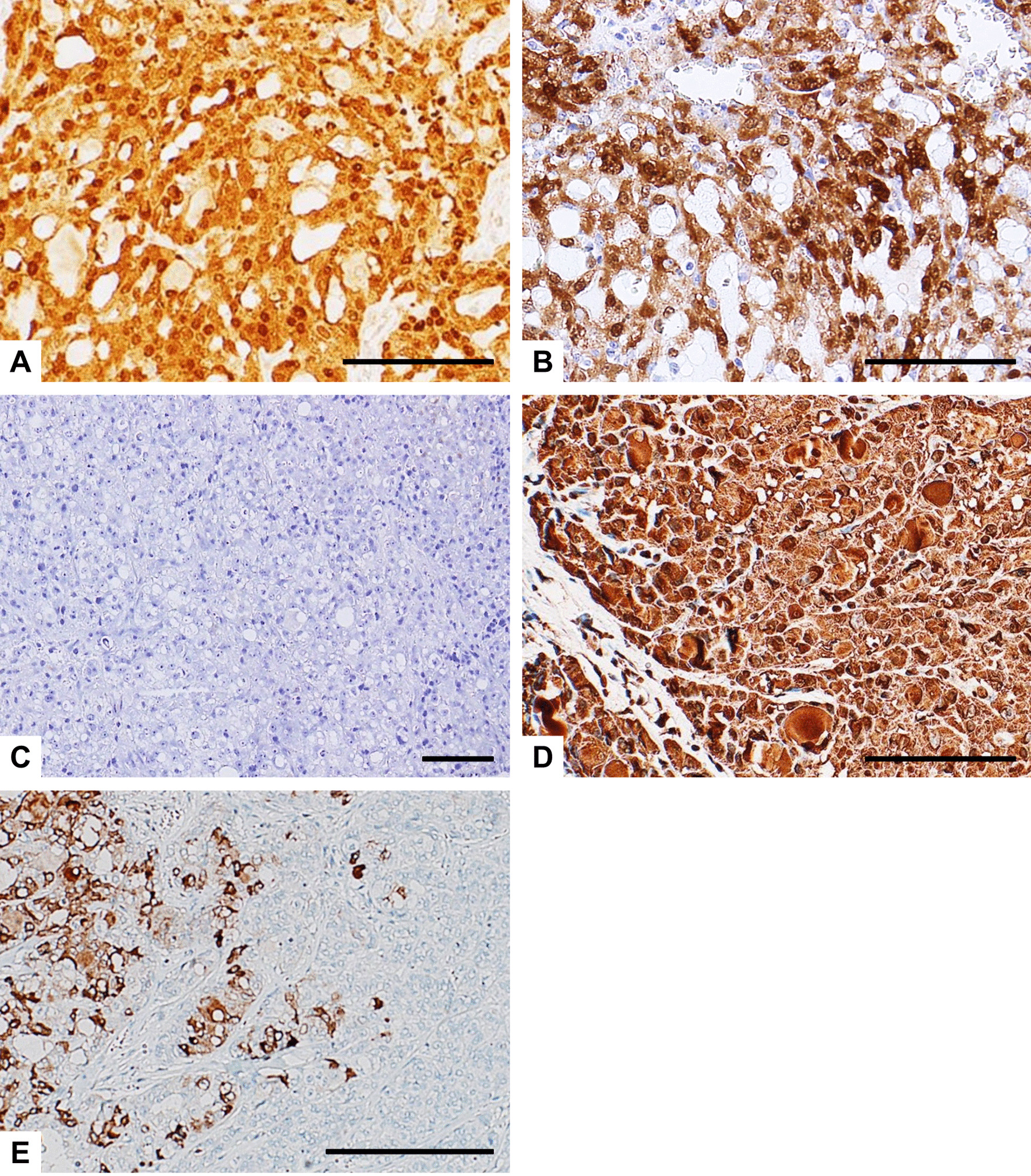
Representative immunohistochemical findings of S-100 protein (**A**–**C**) and mammaglobin (**D** and **E**) in SC cases. The tumor cells show strong and diffuse staining for S-100 protein in Case 1 (**A**) and the initial tumor of Case 3 (**B**), whereas the neoplastic cells in the specimen from the 6th recurrence of Case 3 were negative for S-100 protein (**C**). **D** The tumor cells also show strong and diffuse staining for mammaglobin (Case 1). **E** The HG component displays a low level of mammaglobin expression (right portion), in contrast to a high level of mammaglobin expression in the LG component (left portion, Case 2). Scale bars, 100 µm (**A**–**D**); 500 µm (**E**)

**Fig. 5:**
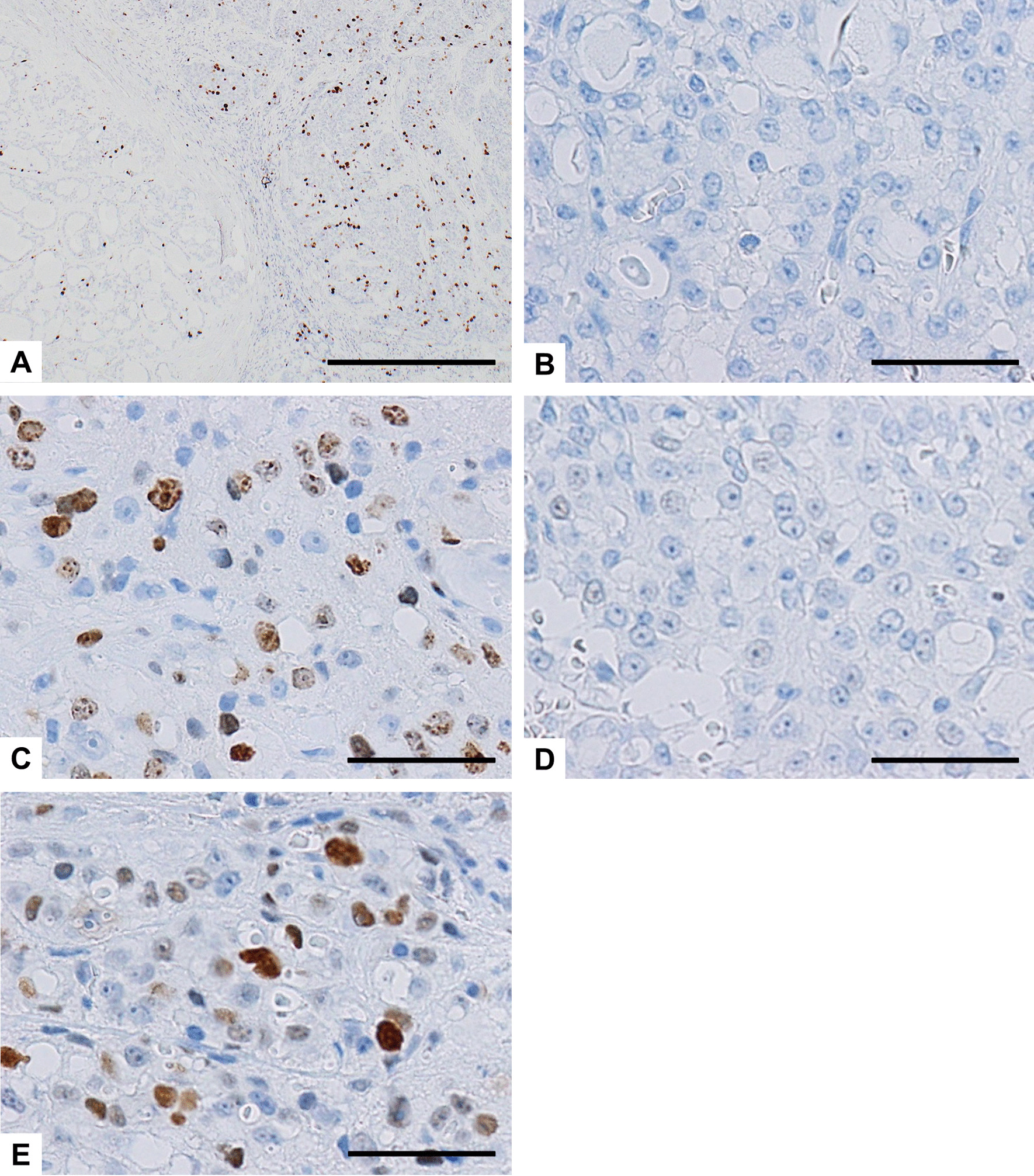
Immunohistochemical findings of Ki-67 in Case 2 (**A**) and Case 3 (**B** and **C**), and p53 (**D** and **E**) in Case 3. **A** The HG component displays a high Ki-67 LI (right portion), in contrast to a low Ki-67 LI in the LG component (left portion, Case 2). **B** and **D**, the initial tumor; **C** and **E**, the specimen from the 6th recurrence. Ki-67 LI (**C**) and the p53-positive cell population (**E**) were markedly higher in the recurrent tumor compared with the initial tumor (**B** and **D**, respectively). Scale bars, 500 µm (**A**); 50 µm (**B**–**E**)

The immunohistochemical findings are summarized in Table 1. The LG and HG components of the tumors in Case 2 and 3 are shown separately. Immunohistochemical analysis revealed that all examined SC cases and components were positive for p-STAT5 (Fig. 3A–D), while all but one component were positive for S-100 protein and mammaglobin, with typically strong and diffuse staining (Fig. 4A–E, respectively). All but one component were also positive for GATA3, while all examined samples were negative for DOG1. These immunohistochemical findings for the 3 cases were consistent with the diagnosis of SC. Notably, the LG and HG components showed different expression patterns of these markers. The proliferative activity of the LG components was low, with the Ki-67 (MIB1) labeling index (LI) ranging from 0 to 8%, while those of the HG components were markedly higher (up to 30% and 32%). In Case 2, the HG component displayed a low level of mammaglobin expression and a high Ki-67 LI, in contrast to a high level of mammaglobin expression and a low Ki-67 LI in the LG component (Figs. 4E and 5A). In Case 3, the Ki-67 LI (Fig. 5B and C) and p53-positive cell populations (Fig. 5D and E) were markedly higher in the specimen taken at the 6th recurrence in comparison with the values for the specimen taken from the initial tumor. These results suggest that the biological characteristics of the tumor changed and the proliferative activity increased due to HGT (dedifferentiation). No staining for p53 was found in Case 1 and 2. All cases and components showed focal membranous staining for EGFR and strong membrane positivity for β-catenin, whereas staining with HER2 was all negative (Table 1). The primary antibodies used are summarized in Additional file 1.

**Table 1:** Immunohistochemical findings in 3 cases of SC

Case#		S-100 protein	p-STAT5	Mammaglobin	GATA3	DOG1	HER2	EGFR	β-Catenin	p53	Ki-67 labeling index (%)
1		+	+	+	−	−	−	+	+	−	8
2	LG	+	+	+	+	−	−	+	+	−	3
	HG	+	+	−	+	−	−	+	+	−	32
3	LG	+	+	+	+	−	−	+	+	−	0
	HG	−	+	+	+	−	−	+	+	+	30

LG, low-grade component; HG, high-grade component

### FISH for ETV6 rearrangement

**Fig. 6:**
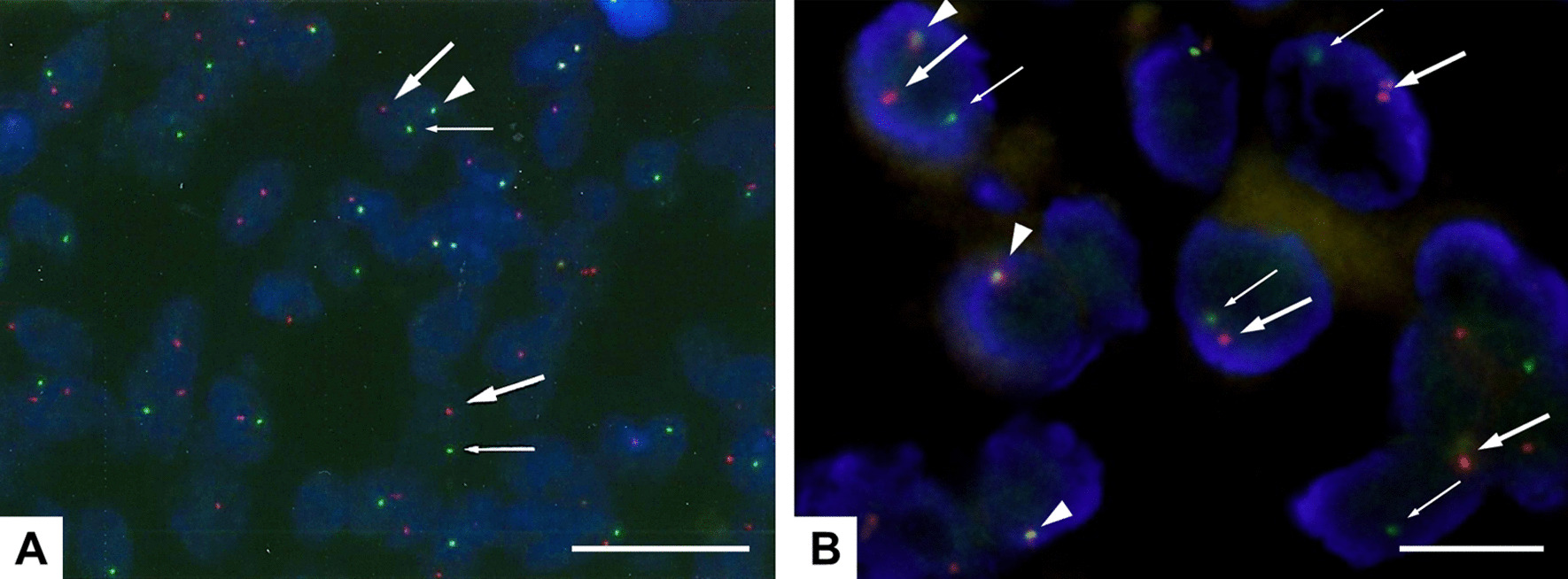
FISH study with an ETV6 (12p13) break apart probe for Case 2 (**A**) and the specimen from the 4th recurrence of Case 3 (**B**). The yellow signal (red/green fusion, arrow head) demonstrates an intact chromosome, whereas a separated red (big arrow) and green signals (small arrow) indicate a break in the ETV6 gene. (**A**) Fig. 3D adapted from Suzuki et al. 2017 [22]. Permission was obtained from Karger AG. Scale bars, 25 µm (**A**); 10 µm (**B**)

A fluorescence in situ hybridization (FISH) study was performed as described previously [23]. Briefly, unstained 4-µm paraffin-embedded tissue sections were put through deparaffinization and protease pretreatment steps before they were denatured and hybridized overnight with a commercial ETV6 (12p13) break apart probe (Vysis ETV6 Break Apart FISH Probe Kit, Abbott) according to the manufacturer’s instructions. Slides were analyzed using AXIO imager M2 (Carl Zeiss Meditec, Germany) and ISIS software (MetaSystems, Germany). The measured resolutions of acquired FISH images were both 96 dpi, and the resolutions of the images were enhanced using GNU Image Manipulation Program (GIMP) software (https://www.gimp.org/). For FISH interpretation, 100 randomly selected nonoverlapping tumor cell nuclei were examined for the presence of yellow (red/green fusion) or green and red fluorescent signals. Yellow signals were considered negative, and separate red and green signals were considered positive [8, 10, 23]. The cutoff value was set at 10%. FISH study with an ETV6 break apart probe revealed split signals in the nuclei in all 3 cases (Fig. 6A and B). These findings supported a diagnosis of SC. The FISH results for Case 2 (Fig. 6A) were reported previously [22].

### Gene fusion analysis

For the NGS-based fusion assay, 6 samples of SC from these 3 cases were subjected to the analysis. For Case 3, we chose 4 samples from different time points (the primary tumor, and 3rd, 5th, and 6th recurrences) to investigate possible gene alterations associated with HGT. The information regarding the 6 samples is shown in Tables 2 and 3. Total nucleic acid (TNA) was isolated from FFPE tumor specimens using a Maxwell® CSC RNA FFPE Kit (Promega, Wisconsin, U.S.A., AS1360) and then used for library preparation using STRATAFIDE RNA (ArcherDX, Colorado, U.S.A.) according to the manufacturers’ protocol, and subsequent NGS was performed using Illumina MiSeqDX instruments and reagents (Illumina, California, U.S.A.). Analysis of sequencing results was performed using the Archer Analysis software v 6.0.3.2 (ArcherDX).

**Table 2:** Details of NGS analysis in 6 samples from 3 cases of SC

Case#	Specimen site	Sample collection date	RNA input (ng)	Gene fusion	No. valid fusion reads	% of reads supporting fusion	No. unique start sites	Breakpoints
1	Parotid	2015	100	*ETV6-NTRK3*	6	10.17	3	chr12:12022903 chr15:88483984
				*NTRK3-ETV6*^†^	8	13.56	3	chr15:88576088 chr12:12037379
2	Lymph node	2016	100	*ETV6-NTRK3*	232	60.42	104	chr12:12022903 chr15:88483984
				*NTRK3-ETV6*^†^	100	49.75	53	chr15:88576088 chr12:12037379
3–1*	Parotid	1994	100	*ETV6-NTRK3*	29	24.17	14	chr12:12022903 chr15:88483984
3–2*	Parotid	2008	100	*ETV6-NTRK3*	14	17.72	9	chr12:12022903 chr15:88483984
3–3*	Parotid	2012	100	*ETV6-NTRK3*	873	57.62	167	chr12:12022903 chr15:88483984
3–4*	Lymph node	2012	100	*ETV6-NTRK3*	288	56.58	104	chr12:12022903 chr15:88483984

*3–1, the initial tumor; 3–2, 3rd recurrence; 3–3, 5th recurrence; 3–4, and 6th recurrence of case 3, respectively

^†^Reciprocal fusion which is secondary and non –driver mutation

**Table 3:** Details of NGS analysis in 6 samples at lower RNA input level

Case#	Specimen site	Sample collection date	RNA input (ng)	Gene fusion	No. valid fusion reads	% of reads supporting fusion	No. unique start sites	Breakpoints
1	Parotid	2015	10	*ETV6-NTRK3*	8	13.56	3	chr12:12022903 chr15:88483984
				*NTRK3-ETV6*^†^	6	10.17	3	chr15:88576088 chr12:12037379
2	Lymph node	2016	10	*ETV6-NTRK3*	212	70.20	96	chr12:12022903 chr15:88483984
				*NTRK3-ETV6*^†^	76	48.41	44	chr15:88576088 chr12:12037379
3–1*	Parotid	1994	10	*ETV6-NTRK3*	23	34.33	10	chr12:12022903 chr15:88483984
3–2*	Parotid	2008	10	*ETV6-NTRK3*	8	15.38	4	chr12:12022903 chr15:88483984
3–3*	Parotid	2012	10	*ETV6-NTRK3*	779	55.25	162	chr12:12022903 chr15:88483984
3–4*	Lymph node	2012	10	*ETV6-NTRK3*	255	72.24	97	chr12:12022903 chr15:88483984

*3–1, the initial tumor; 3–2, 3rd recurrence; 3–3, 5th recurrence; 3–4, and 6th recurrence of case 3, respectively

^†^Reciprocal fusion which is secondary and non –driver mutation

The pre-analytic quality control metrics for sample input prior to sequencing were performed by Archer PreSeq RNA QC Assay (ArcherDX). The following are the suggested acceptance criteria for these parameters: RNA Extraction ≥ 1.0 ng/uL; PreSeq QC Assay: |ΔC_q_|< 12; and Sample Library Quantification > 4 nM. All samples passed the acceptance criteria for RNA concentration and sample library quantification. Sequencing cluster density was 924 K/mm^2^ and exceeded the recommended instrument minimum (400 K clusters/mm^2^). Percent ≥ Q30 (a measure of sequencing quality) was 96.14% and exceeded the manufacturer’s recommendations (≥ 80% of bases with > Q30).

All 6 samples were tested initially with an RNA input of 100 ng and were identified to contain *ETV6-NTRK3* fusion (Table 2). Analysis of the data by Archer Analysis v 6.0.3.2 categorized them as strong fusions and they were cross-checked against Archer Quiver Fusion Database (http://quiver.archerdx.com). All 6 samples were subsequently tested at lower RNA input levels (ranging from 10 to 90 ng) and sequencing data were analyzed with STRATAFIDE RNA software v 1.12.2 (ArcherDX, IVD software version). Even at the lowest input level (at 10 ng of RNA input), libraries prepared from the 6 samples demonstrated the presence of strong *ETV6-NTRK3* fusion (Table 3). Two of these samples showed the presence of reciprocal fusion *NTRK3-ETV6* which is considered to be a secondary and non-driver mutation. The breakpoints involved in *NTRK3-ETV6* (chr15:88576088, chr12:12037379) differed from the NTRK3 and ETV6 gene breakpoints involved in the driver mutation *ETV6-NTRK3* (chr12:12022903, chr15:88483984). The patient samples did not show the presence of any other known or novel fusions. Details of the sequencing metrics for the samples analyzed in this study are listed in Tables 2 and 3. All sequencing metrics indicating the quality of the samples and libraries are listed in Additional file 2. The unfiltered fusions and true fusions observed after application of filters to identify only the true and strong fusions are listed in Additional file 3 and Additional file 4, respectively.

## Discussion and conclusions

Generally, the clinical course of SC of the salivary gland is characterized by a moderate risk of local recurrence and a low risk of distant metastases. On the basis of the few cases with follow-up data reported, SC is currently regarded as a LG carcinoma with an overall favorable prognosis [1, 24–27]. However, a small subset of SCs has the potential for regional and distant metastasis [4]. To our knowledge, 12 cases (including the current cases) of SCs with HGT of the head and neck have been reported [10, 17, 28–31]. In this study, we report a clinicopathological and molecular genetic study of 3 cases of SC, including a patient with conventional LG SC, and 2 cases with HGT. One patient with biphasic tumor histology consisting of both LG and HG components showed a dramatic clinical response to the pan-TRK inhibitor, entrectinib, for the treatment of locoregional recurrence and pulmonary metastasis. The other patient with HGT (Case 3) is the first case of SC, which was initially diagnosed as LG carcinoma and later developed into HG-transformed SC after multiple recurrences over a long time period.

HGT is defined as the histologic progression of a LG malignant neoplasm to a HG histology that lacks the original distinct pathological characteristics [10, 11]. The conventional and HG carcinomatous areas are clearly distinguished, although a transitional zone can be identified in some cases [11]. The HG component is characterized by pleomorphism, necrosis and a high cell proliferation rate, as assessed by mitotic count and Ki-67 LI [11]. Unlike conventional SC, HG-transformed SC is a much more aggressive tumor that follows an aggressive clinical course, resulting in local recurrences, cancer dissemination, and death [10], as seen in Case 3 of our series. In view of the aggressive nature of HG-transformed SC, radical surgery and adjuvant radiotherapy are recommended for the management of such patients [10]. In addition, the resected specimens of all SCs should be thoroughly evaluated to avoid missing any HG components, particularly in cases of recurrence. Failure to recognize areas of HGT may result in therapeutic mismanagement of the patient [10]. As previously reported [10], we define the HG component of SC using conventional histomorphologic criteria, such as nuclear polymorphism, distinctive nucleoli, increased mitotic activity, increased Ki-67 LI, and areas of necrosis, and these parameters are correlated with clinical outcome. As expected, we confirmed that the Ki-67 LI was higher in the HG components than in the LG components, suggesting that the proliferation activity of tumor cells was much higher in HG-transformed SCs. In HG-transformed salivary gland cancer, the LG and HG components often show different immunohistochemical expression patterns, reflecting the “dedifferentiation” [11]. Skálová et al. described that the HG component of SCs revealed strong membrane staining for EGFR and β-catenin [10]. p53 abnormalities and amplification of HER2 have also been demonstrated in HG-transformed salivary gland cancer [11, 14, 18]. In our series, the difference of staining positivity for EGFR, β-catenin and HER2 between LG and HG components was not observed. Only one HG component in the patient with repeated recurrence showed positive staining for p53. Whether p53 abnormalities cause HGT in SC needs further investigation.

Immunohistochemically, SC typically shows positive staining for S-100 protein and mammaglobin [1]. SC may also express GATA3 [32] and p-STAT5 [23], whereas DOG1 stain is positive in the majority of cases of AcCC [33]. In this study, 4 of 5 examined components were positive for S-100 protein, mammaglobin and GATA3. By contrast, all SC cases and components examined in our series were positive for p-STAT5, suggesting that p-STAT5 is a reliable marker for SC irrespective of its histological grade. Similarly, Kawahara et al. reported that p-STAT5 has comparable sensitivity and higher specificity for the detection of SC of the salivary gland than does mammaglobin in terms of both immunohistochemistry and immunocytochemistry [23]. DOG1 expression was all negative in our series and was useful to exclude AcCC as a differential diagnosis.

Recognizing SC and testing for *ETV6-NTRK3* gene rearrangement are valuable for patient treatment, particularly in cases of HG-transformed SC, as the presence of *ETV6-NTRK3* translocation may represent a therapeutic target [6, 7, 19, 20]. Entrectinib, the pan-TRK, ROS1, and ALK inhibitor, demonstrated its efficacy and feasibility for the treatment of patients harboring gene rearrangements in three clinical trials (ALKA-372-001, STARTRK-1, and STARTRK-2) [6, 21]. Herein, we presented a patient with HG-transformed SC successfully treated with entrectinib without any serious adverse events. The *ETV6-NTRK3* translocation has been detected not only in secretory carcinomas, but also in most cases of infantile fibrosarcomas, congenital mesoblastic nephromas [34], chronic eosinophilic leukemias [35], acute myeloid leukemias [36], and some papillary carcinomas of the thyroid with and without previous irradiation [37]. Patients with these diseases harboring the *ETV6-NTRK3* translocation may also gain some benefit from treatment with entrectinib.

Although *ETV6-NTRK3* gene fusion is canonical for SC, advances in molecular profiling of this tumor have led to the discovery of novel ETV6 fusion partners, such as RET [8], MET [38], and MAML3 [39]. These alternative translocations appear to exhibit HG histology and more aggressive biological features [8, 38, 39]. Of note, *ETV6-NTRK3* gene rearrangement can benefit from NTRK3-targeted therapy; whereas, there is no precedent for using these drugs in cases lacking NTRK3 fusions [38]. SC with these alternative translocations is extremely rare; therefore, it is conceivable that other molecular pathways for the progression of HG-transformed SC exist. To date, the pathogenesis of HGT of salivary gland carcinomas is not well understood [10]. In this study, a targeted NGS-based fusion assay demonstrated that all of the 6 clinical samples of SC showed the presence of the *ETV6-NTRK3* fusion transcripts. Additionally, 2 of the 6 samples (2 of 3 SC cases) showed the presence of reciprocal *NTRK3-ETV6* gene fusion. The fusion supporting metrics for secondary fusion were weaker than those for the driver mutation. To our knowledge, only one study has described the reciprocal *NTRK3-ETV6* fusion transcript in SC of the salivary gland [40]. Whether this reciprocal transcript has a biological function requires elucidation.

This study has some limitations. First, the number of samples in this study was small. Second, samples analyzed in this study were collected over a long period (from 1994 to 2012), which might affect the quality of the samples for immunohistochemical and molecular genetic study. It is worth emphasizing that HGT of SC is a rare condition, and the transition of clinical and pathological findings over a long time period observed in the extremely rare case, which progressed from LG carcinoma to HG-transformed SC, provides potentially useful information with regard to this patient population. Regarding the quality of the samples for immunohistochemistry, previous reports showed that most antigens in stored FFPE blocks are well preserved at least for several decades [41, 42]. Indeed, positive staining for immunohistochemical markers such as S-100 protein, p-STAT5, mammaglobin, GATA3, EGFR and β-catenin was clearly observed even in the oldest sample in this study. In the NGS-based fusion assay, all of the 6 clinical samples showed the presence of the *ETV6-NTRK3* fusion at the same breakpoint (chr12:12022903, chr15:88483984), even when tested with multiple variables examined independently (such as testing at various RNA input amounts, different time points, and analysis by different analysis software). Thus, the quality of the older samples did not affect the effective evaluation and positive identification of strong aberrations or RNA fusion (like *ETV6-NTRK3* fusion). Our gene fusion analysis did not show the presence of any other known or novel fusions. Further investigation is required to identify the molecular genetic pathogenesis of HGT in SC.

In conclusion, we have described the histologic, immunohistochemical, and molecular genetic findings of 3 SC cases with different clinical characteristics. HG-transformed SC showed aggressive clinical and pathological features with increased Ki-67 LI. Targeted treatments for the gene rearrangement could be an option for HG-transformed SC in addition to radical surgery and radiotherapy.

## Supplementary Information

**Additional file 1.** Antibodies used for immunohistochemical study.

**Additional file 2.** All sequencing metrics indicating the quality of the samples and libraries.

**Additional file 3.** All the unfiltered fusions before application of filters to identify strong and true fusions.

**Additional file 4.** List of true fusions observed after application of filters to identify only the true and strong fusions.

## Data Availability

The datasets analyzed during the current study are available in the DDBJ Sequenced Read Archive (https://ddbj.nig.ac.jp/search), under the accession numbers DRR331632-DRR331637.

## Abbreviations

SC: Secretory carcinoma
AcCC: Acinic cell carcinoma
FISH: Fluorescent in situ hybridization
RT-PCR: Reverse transcriptase polymerase chain reaction
FFPE: Formalin-fixed paraffin-embedded
HGT: High-grade transformation
LG: Low-grade
NGS: Next-generation sequencing
LI: Labeling index
TNA: Total nucleic acid
