# Supplementary material for: Clinicopathological investigation of secretory carcinoma cases including a successful treatment outcome using entrectinib for high-grade transformation: a case report
Source: BMC Med Genomics. 2022 Jan 6;15:6. doi: 10.1186/s12920-022-01155-6 (PMC8739673; doi:10.1186/s12920-022-01155-6)
Supplement: Supplementary file 1 — Additional file 1. Antibodies used for immunohistochemical study. [file 12920_2022_1155_MOESM1_ESM.docx]

**Additional file 1.** Antibodies used for immunohistochemical study

| Antibody specificity | Clone | Source |
| --- | --- | --- |
| S-100 protein | polyclonal | Dako |
| p-STAT5 | C71E5 | Cell Signaling Technology |
| mammaglobin | 304-1A5 | Dako |
| GATA3 | L50-823 | Roche |
| DOG1 | SP31 | Roche |
| HER2 | 4B5 | Roche |
| EGFR | A-10 | Santa Cruz |
| β-catenin | 14/Beta-Catenin | BD Biosciences |
| p53 | DO-7 | Dako |
| Ki-67 | MIB-1 | Dako |
